# Immune checkpoint protein PD-L1 promotes transcription of angiogenic and oncogenic proteins IL-8, Bcl3, and STAT1 in ovarian cancer cells

**DOI:** 10.1016/j.jbc.2025.108339

**Published:** 2025-02-22

**Authors:** Suprataptha U. Reddy, Rachel Sham, Khalani Smith, Bijaya Gaire, Ales Vancura, Ivana Vancurova

**Affiliations:** Department of Biological Sciences, St John's University, New York, New York, USA

**Keywords:** nuclear PD-L1, transcription, IFNγ, immune checkpoint blockade, cancer immunotherapy, ovarian cancer, RNA polymerase II, tumor immunology

## Abstract

Immunotherapies blocking cell surface signaling of the immune checkpoint PD-L1 have shown great promise in several cancers, but the results have been disappointing in ovarian cancer (OC). One of the main underlying mechanisms likely consists of the cell-intrinsic intracellular functions of PD-L1, which are incompletely understood. The expression of PD-L1 in OC cells is induced by interferon-**γ** (IFN**γ**), a pleiotropic cytokine produced in response to chemotherapy or immune checkpoint blockade. We have recently shown that IFNγ induces expression of the proto-oncogene Bcl3, the proangiogenic chemokine interleukin-8 (IL-8)–CXCL8, and the transcription factor STAT1, resulting in increased OC cell proliferation and migration. Here, we report that IFN**γ**-induced expression of PD-L1 results in PD-L1 recruitment to IL-8, Bcl3, and STAT1 promoters. The occupancy of PD-L1 at IL-8, Bcl3, and STAT1 promoters is associated with increased histone acetylation and RNA polymerase II recruitment to these promoters. Suppression of IFN**γ**-induced PD-L1 decreases the expression of IL-8, Bcl3, and PD-L1 and increases apoptosis in OC cells. Together, these findings demonstrate that PD-L1 promotes transcription of IL-8, Bcl3, and STAT1, thus providing a novel function of PD-L1 in cancer cells, and suggesting that the increased IL-8, Bcl3, and STAT1 expression mediated by PD-L1 might contribute to the limited effectiveness of cancer immunotherapies targeting the surface expression of PD-L1 in OC.

The immune checkpoint protein PD-L1 (CD-274, B7-H1) was originally discovered as a cell membrane protein that binds to its receptor PD-1 expressed on T cells, thus inhibiting immune responses, and promoting immune escape (1, 2, 3, 4, 5). However, more recent studies demonstrated also intracellular localization of PD-L1, which has important nonimmune, cell-intrinsic functions, including increased cancer cell proliferation, cell cycle progression, cell survival, mechanistic target of rapamycin signaling, DNA damage response, and development of drug resistance (6, 7, 8, 9, 10, 11, 12, 13, 14, 15, 16, 17, 18, 19, 20). The intracellular PD-L1 has been detected both in the cytoplasm and in the nucleus, where its increased levels have been associated with increased chemoresistance and cancer progression (21, 22, 23, 24, 25, 26, 27, 28). However, the specific nuclear functions of PD-L1 in cancer cells are just becoming understood.

Ovarian cancer (OC) is among the leading causes of cancer deaths among women, with a high incidence of relapse and drug resistance and low survival rates. Even though many OC tumors express significant levels of PD-L1, immunotherapies blocking cell surface PD-L1–PD-1 interaction in OC patients have produced unsatisfactory results (9, 20, 29, 30), but the underlying mechanisms are largely unknown. A better understanding of the intracellular, and particularly of the nuclear, functions of PD-L1 is essential for the development of more effective PD-L1 targeting combination anticancer therapies.

The expression of cell surface PD-L1 on cancer cells is induced by interferon-γ (IFNγ), a pleiotropic cytokine produced not only by activated T cells but also in response to chemotherapy or immune checkpoint blockade used in cancer treatment (30, 31, 32, 33). We have recently shown that IFNγ induces expression of the antiapoptotic proto-oncogene and transcriptional regulator Bcl3, which then promotes transcription of PD-L1 and the proangiogenic and antiapoptotic chemokine interleukin-8 (IL-8)–CXCL8 in OC cells (34, 35, 36, 37, 38). The IFNγ-induced Bcl3, IL-8, and PD-L1 expression is dependent on STAT1 signaling and results in increased proliferation and migration of OC cells (34, 35, 36, 37, 38).

By investigating the mechanisms by which IFNγ induces PD-L1 expression, we have found that the IFNγ-induced PD-L1 expression leads to a partial localization of PD-L1 in the nucleus in OC cells. Our results show that the nuclear PD-L1 promotes transcription of the antiapoptotic genes IL-8, Bcl3, and STAT1, and suppression of PD-L1 increases apoptosis in OC cells. The mechanisms by which PD-L1 promotes transcription of IL-8, Bcl3, and STAT1 involve RNA polymerase II (pol II) recruitment and increased promoter acetylation. Intriguingly, our results show that PD-L1 itself is recruited to IL-8, Bcl3, and STAT1 promoters in IFNγ-stimulated OC cells. These findings reveal a novel function of PD-L1 in cancer cells and indicate that the IFNγ-induced expression of IL-8, Bcl3, and STAT1, mediated by nuclear PD-L1, might contribute to the limited effectiveness of cancer immunotherapies targeting the surface expression of PD-L1.

## Results

### IFN**γ**-induced PD-L1 expression leads to a partial nuclear localization of PD-L1 in OC cells

**Figure 1:**
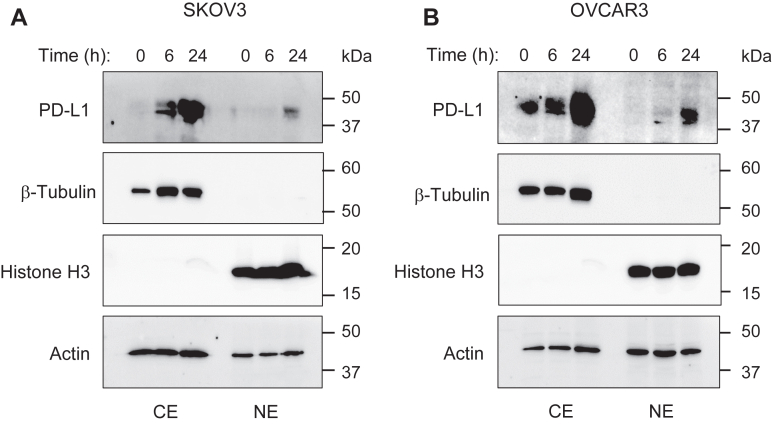
**IFNγ induces PD-L1 expression that leads to a partial nuclear localization of PD-L1 in OC cells.** (*A*) SKOV3 and (*B*) OVCAR3 cells were incubated with 50 ng/ml IFNγ for the indicated times, and CE and NE extracts were prepared and analyzed by immunoblotting using PD-L1 antibody that recognizes endogenous PD-L1. The purity of cytoplasmic and nuclear fractions was monitored by using histone H3 and β-tubulin antibodies. Each lane represents approximately 10^5^ cells; equal protein loading was confirmed by using actin antibody. The experiment was repeated at least three times; representative images are shown. CE, cytoplasmic extract; IFNγ, interferon-γ; NE, nuclear extract; OC, ovarian cancer.

Nuclear accumulation of PD-L1 has been associated with increased chemoresistance and tumorigenesis in cancer cells (21, 22, 23, 24, 25, 26, 27, 28), but little is known about the factors that induce the nuclear localization of PD-L1. To investigate whether the IFNγ-induced expression of PD-L1 might alter its subcellular localization in OC cells, we prepared cytoplasmic extract and nuclear extract from IFNγ-stimulated SKOV3 and OVCAR3 cells and analyzed the cytoplasmic and nuclear levels of PD-L1 by immunoblotting. For PD-L1 detection, we used PD-L1 E1L3N monoclonal antibody, which specifically recognizes the endogenous PD-L1 protein (39, 40). As shown in Figure 1, unstimulated OC cells expressed only low levels of PD-L1, which was localized mainly in the cytoplasm, and IFNγ rapidly induced PD-L1 expression in the cytoplasm in both SKOV3 (Fig. 1*A*) and OVCAR3 (Fig. 1*B*) cells. Intriguingly, IFNγ also increased PD-L1 levels in the nucleus in SKOV3 and OVCAR3 cells, particularly in cells stimulated with IFNγ for 24 h (Fig. 1), indicating that increased expression of PD-L1 leads to a translocation of a substantial fraction of PD-L1 to the nucleus in OC cells.

### PD-L1 suppression increases apoptosis in IFN**γ**-stimulated OC cells

**Figure 2:**
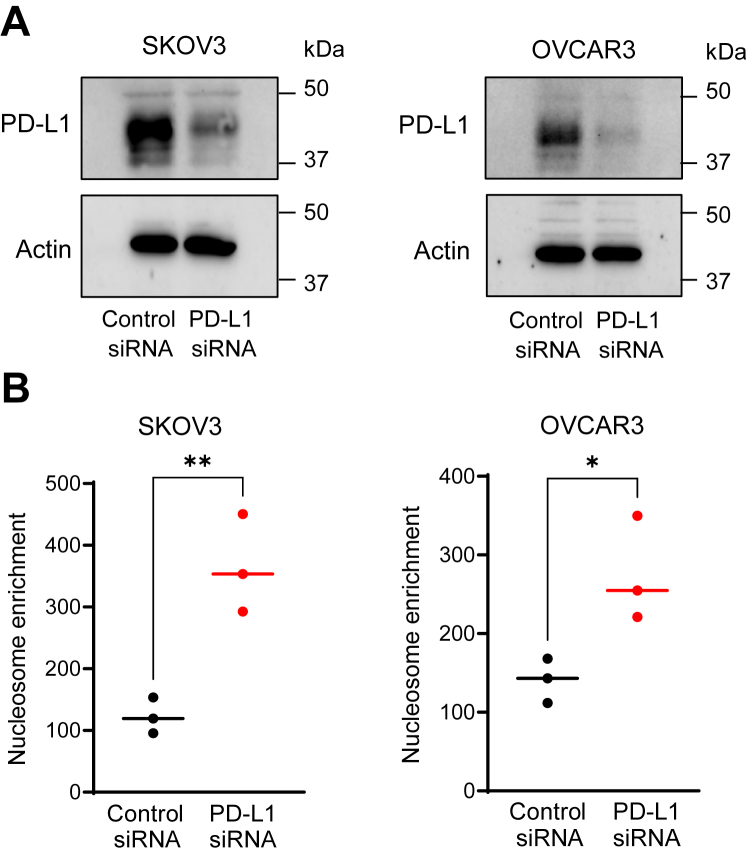
**PD-L1 suppression increases apoptosis in IFNγ-stimulated OC cells**. *A*, Western blotting of PD-L1 and control actin in whole-cell extracts (WCEs) prepared from SKOV3 and OVCAR3 cells transfected with control and PD-L1 siRNA and stimulated 24 h with IFNγ (50 ng/ml). Each lane represents approximately 10^4^ cells; equal protein loading was confirmed using actin antibody. *B*, apoptosis measured by cytoplasmic nucleosome enrichment assay in SKOV3 and OVCAR3 cells transfected with control or PD-L1 siRNA and stimulated with IFNγ (24 h, 50 ng/ml). Results represent three independent experiments and are presented as the mean ± SD (n = 3). The statistical significance was evaluated by unpaired *t* test; ∗*p* < 0.05; ∗∗*p* < 0.01, compared with cells transfected with control siRNA. IFNγ, interferon-γ; OC, ovarian cancer.

To elucidate the function of IFNγ-induced PD-L1, we first analyzed the effect of PD-L1 suppression on apoptosis in IFNγ-stimulated OC cells. SKOV3 and OVCAR3 cells were transfected with PD-L1 or control siRNA, stimulated with IFNγ (50 ng/ml, 24 h), and apoptosis was evaluated by measuring the release of nucleosomes into the cytoplasm. As shown in Figure 2*A*, PD-L1 suppression by siRNA substantially decreased the total cellular levels of PD-L1 in IFNγ-stimulated SKOV3 and OVCAR3 cells compared with cells transfected with control siRNA. Importantly, PD-L1 suppression significantly increased apoptosis in IFNγ-stimulated SKOV3 and OVCAR3 cells (Fig. 2*B*), suggesting that PD-L1 might promote expression of antiapoptotic genes in OC cells.

### PD-L1 suppression downregulates the expression of IL-8, Bcl3, and STAT1 in IFN**γ**-stimulated OC cells

**Figure 3:**
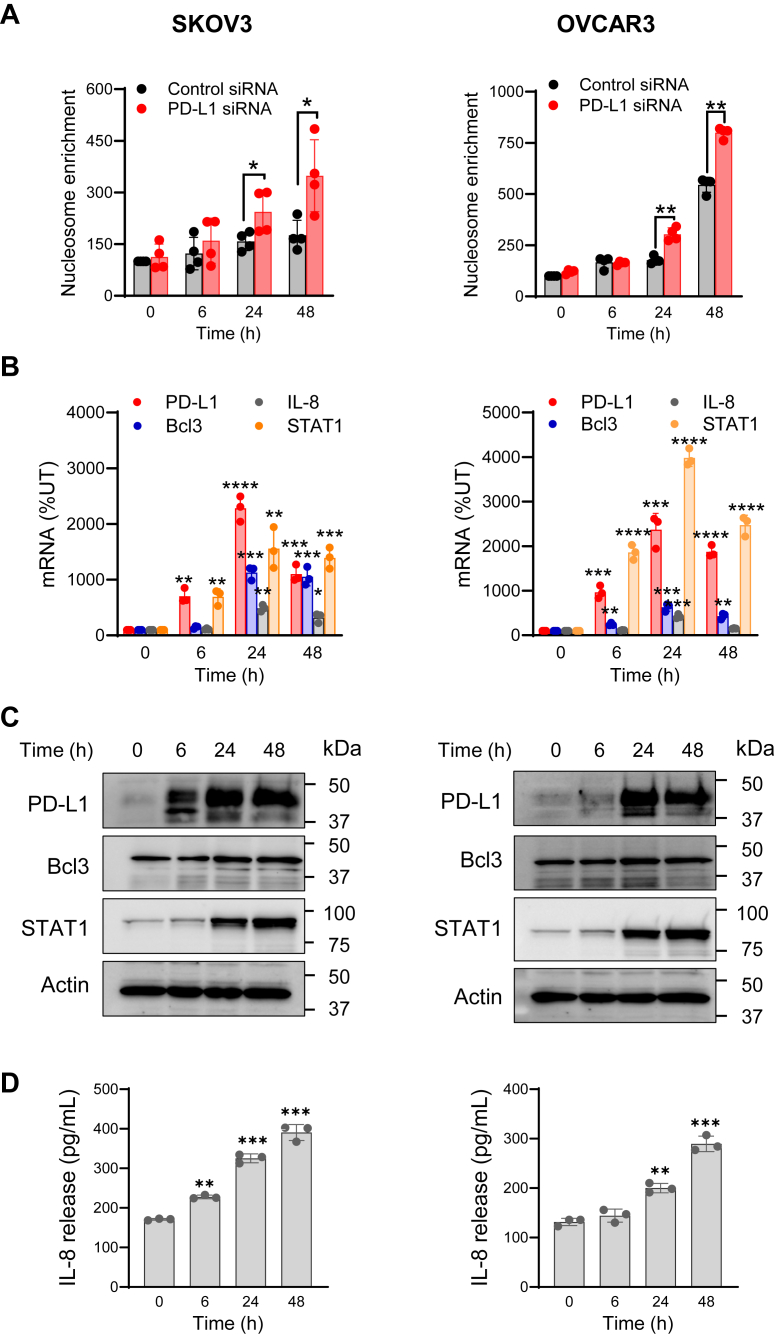
**Kinetics of IFNγ-induced apoptosis and IL-8, Bcl3, and STAT1 expression in IFNγ-stimulated OC cells.***A*, apoptosis measured by cytoplasmic nucleosome enrichment assay in SKOV3 and OVCAR3 cells transfected with control or PD-L1 siRNA and stimulated with IFNγ (50 ng/ml). Data are presented as the mean ± SD (n = 4). Statistical significance was evaluated by unpaired *t* test; ∗*p* < 0.05; ∗∗*p* < 0.01, compared with cells transfected with control siRNA. *B*, relative mRNA expression of PD-L1, IL-8, Bcl3, and STAT1 analyzed by qRT–PCR in SKOV3 and OVCAR3 cells stimulated with 50 ng/ml IFNγ. Data are presented as the mean ± SD (n = 3). Statistical significance was evaluated by unpaired *t* test; ∗*p* < 0.05; ∗∗*p* < 0.01; ∗∗∗*p* < 0.001; ∗∗∗∗*p* < 0.0001 compared with unstimulated cells. *C*, Western blotting of PD-L1, Bcl3, and STAT1 in WCE prepared from SKOV3 and OVCAR3 cells stimulated with IFNγ (50 ng/ml). The experiment was repeated at least three times; representative images are shown. Each lane represents approximately 5 × 10^4^ cells; equal protein loading was confirmed using actin antibody. *D*, IL-8 release measured by ELISA in supernatants of SKOV3 and OVCAR3 cells stimulated with 50 ng/ml IFNγ. Data are presented as the mean ± SD (n = 3). Statistical significance was evaluated by unpaired *t* test; ∗∗*p* < 0.01; ∗∗∗*p* < 0.001 compared with unstimulated cells. IFNγ, interferon-γ; IL-8, interleukin-8; OC, ovarian cancer; qRT–PCR, quantitative RT–PCR; WCE, whole-cell extract.

We have recently shown that IFNγ induces expression of the antiapoptotic genes, IL-8 and Bcl3, resulting in increased proliferation of OC cells (35, 36, 37, 38). The expression of IL-8 and Bcl3 in IFNγ-stimulated OC cells is regulated by the transcription factor STAT1, the expression of which is also induced by IFNγ (35, 36, 37, 38). To investigate whether the increased apoptosis in IFNγ-stimulated cells with suppressed PD-L1 expression (Fig. 2) might be mediated by IFNγ-induced IL-8, Bcl3, and STAT1, we first examined whether the antiapoptotic effect of PD-L1 in IFNγ-stimulated OC cells correlates with the kinetics of IL-8, Bcl3, and STAT1 expression. As shown in Figure 3*A*, PD-L1 suppression increased apoptosis in SKOV3 and OVCAR3 cells stimulated with IFNγ for 24 and 48 h; this is consistent with the appearance of PD-L1 in the nucleus 24 h after IFNγ stimulation in these cells (Fig. 1). In agreement with previous studies (35, 36, 37, 38), the expression of PD-L1 and STAT1 was low in unstimulated OC cells but was highly induced by IFNγ signaling (Fig. 3, *B* and *C*). In contrast, even unstimulated OC cells expressed Bcl3 and released basal levels of IL-8; the expression of both genes was further increased by IFNγ stimulation for 24 and 48 h (Fig. 3, *B*–*D*).

**Figure 4:**
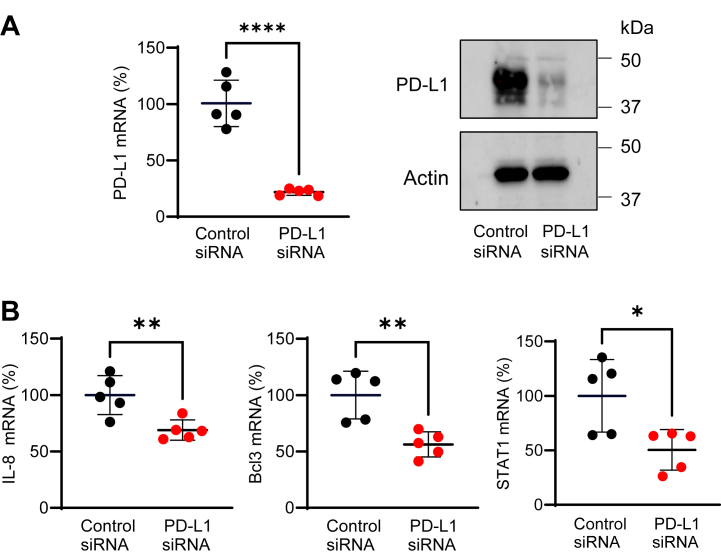
**PD-L1 suppression downregulates mRNA levels of IL-8, Bcl3, and STAT1 in IFNγ-stimulated SKOV3 cells.***A*, relative PD-L1 gene expression analyzed by qRT–PCR (*left*) and protein PD-L1 levels analyzed by Western blotting (*right*) confirming PD-L1 suppression in SKOV3 cells transfected with PD-L1 and control siRNA and stimulated with IFNγ (24 h, 50 ng/ml). *B*, relative gene expression of IL-8, Bcl3, and STAT1 analyzed by qRT–PCR in SKOV3 cells transfected with PD-L1 and control siRNA and stimulated with IFNγ as aforementioned. Data are presented as the mean ± SD (n = 5). The statistical significance was evaluated by unpaired *t* test; ∗*p* < 0.05; ∗∗*p* < 0.01; ∗∗∗∗*p* < 0.0001 compared with cells transfected with control siRNA. IFNγ, interferon-γ; IL-8, interleukin-8; qRT–PCR, quantitative RT–PCR.

**Figure 5:**
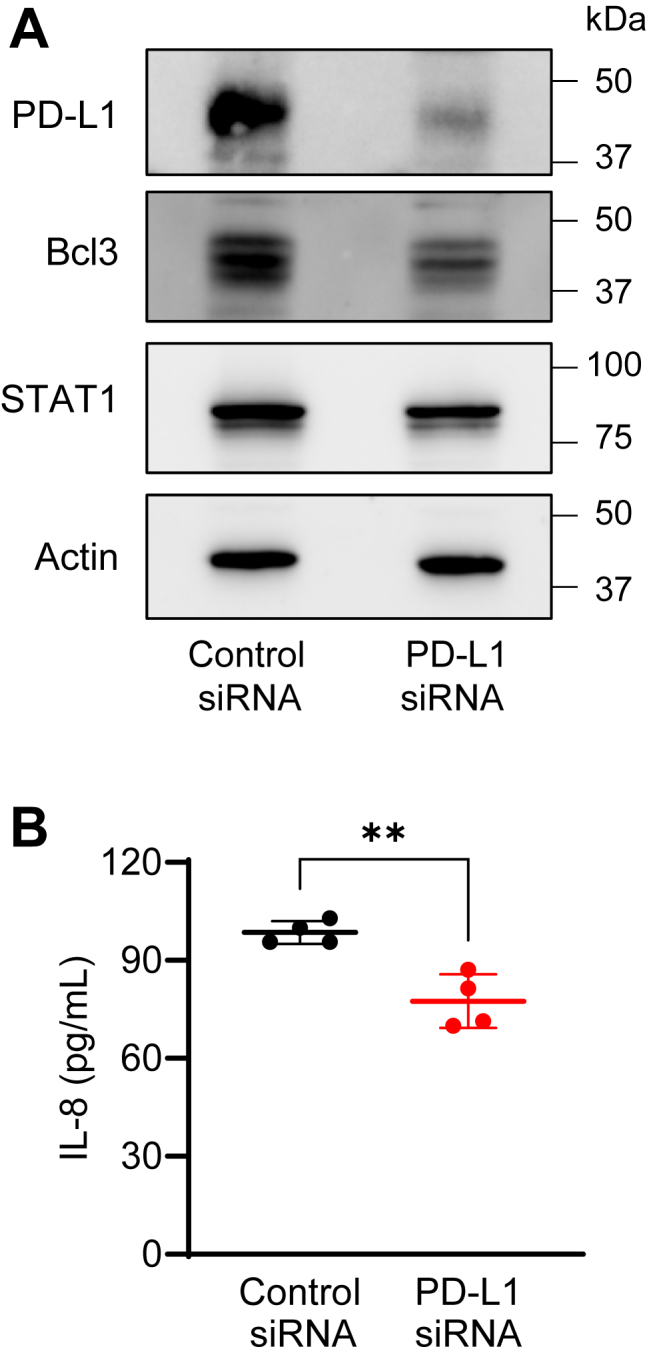
**PD-L1 suppression downregulates protein levels of Bcl3 and STAT1, and IL-8 cytokine release in IFNγ-stimulated SKOV3 cells.***A*, Western blotting of PD-L1, Bcl3, and STAT1 in SKOV3 cells transfected with PD-L1 and control siRNA and stimulated with IFNγ (24 h, 50 ng/ml). Each lane represents approximately 10^4^ cells; equal protein loading was confirmed using actin antibody. *B*, IL-8 release measured by ELISA in supernatant of SKOV3 cells transfected with PD-L1 and control siRNA and stimulated with IFNγ as aforementioned. Data are presented as the mean ± SD (n = 4). The statistical significance was evaluated by unpaired *t* test; ∗∗*p* < 0.01. IFNγ, interferon-γ; IL-8, interleukin-8.

**Figure 6:**
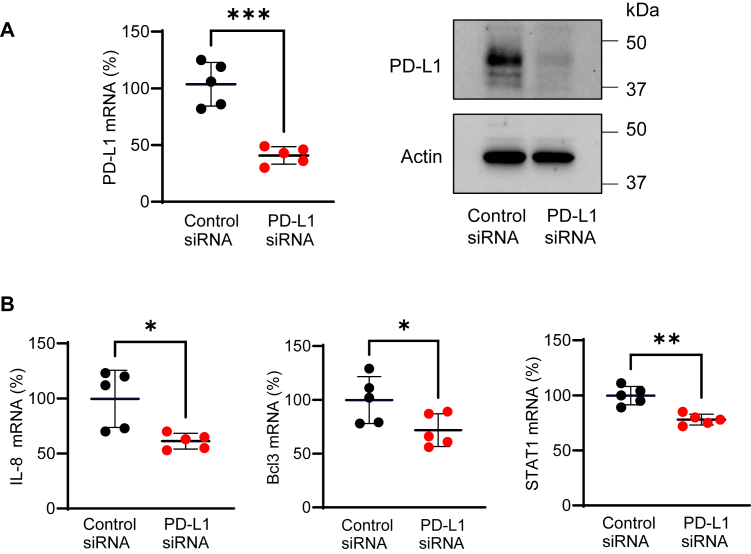
**PD-L1 suppression downregulates mRNA levels of IL-8, Bcl3, and STAT1 in IFN**γ**-stimulated OVCAR3 cells.***A*, relative PD-L1 gene expression analyzed by qRT–PCR (*left*) and PD-L1 protein levels analyzed by Western blotting (*right*) confirming suppression of PD-L1 in OVCAR3 cells transfected with PD-L1 siRNA and stimulated with IFNγ (24 h, 50 ng/ml). *B*, relative gene expression of IL-8, Bcl3, and STAT1 analyzed by qRT–PCR in OVCAR3 cells transfected with PD-L1 and control siRNA and stimulated with IFNγ. Data are presented as the mean ± SD (n = 5). Statistical significance was evaluated by unpaired *t* test; ∗*p* < 0.05; ∗∗*p* < 0.01; ∗∗∗*p* < 0.001 compared with cells transfected with control siRNA. IFNγ, interferon-γ; IL-8, interleukin-8; qRT–PCR, quantitative RT–PCR.

Since the expression of IL-8, Bcl3, and STAT1 was highly induced by 24 h IFNγ stimulation (Fig. 3, *B*–*D*), we hypothesized that the apoptosis induced by PD-L1 suppression in 24 h IFNγ-stimulated OC cells (Fig. 2) is mediated by PD-L1-regulated expression of IL-8, Bcl3, and STAT1. To test this hypothesis, we analyzed mRNA levels of IL-8, Bcl3, and STAT1 in OC cells transfected with control and PD-L1 siRNA and stimulated with IFNγ for 24 h. As shown in Figure 4, PD-L1 suppression significantly decreased the mRNA levels of IL-8, Bcl3, and STAT1 in IFNγ-stimulated SKOV3 cells. In addition, PD-L1 suppression decreased the total protein levels of Bcl3 and STAT1 analyzed in whole-cell extracts of IFNγ-stimulated SKOV3 cells by immunoblotting (Fig. 5*A*) as well as the IL-8 release in cell culture supernatants measured by ELISA (Fig. 5*B*). PD-L1 suppression also significantly decreased the mRNA levels of IL-8, Bcl3, and STAT1 in IFNγ-stimulated OVCAR3 cells (Fig. 6). Together, these results indicated that the cell-intrinsic PD-L1 inhibits apoptosis in IFNγ-stimulated OC cells by increasing the gene expression of IL-8, Bcl3, and STAT1.

### Ectopic expression of PD-L1 induces nuclear localization of PD-L1 and expression of IL-8, Bcl3, and STAT1 in OC cells even in the absence of IFN**γ**

**Figure 7:**
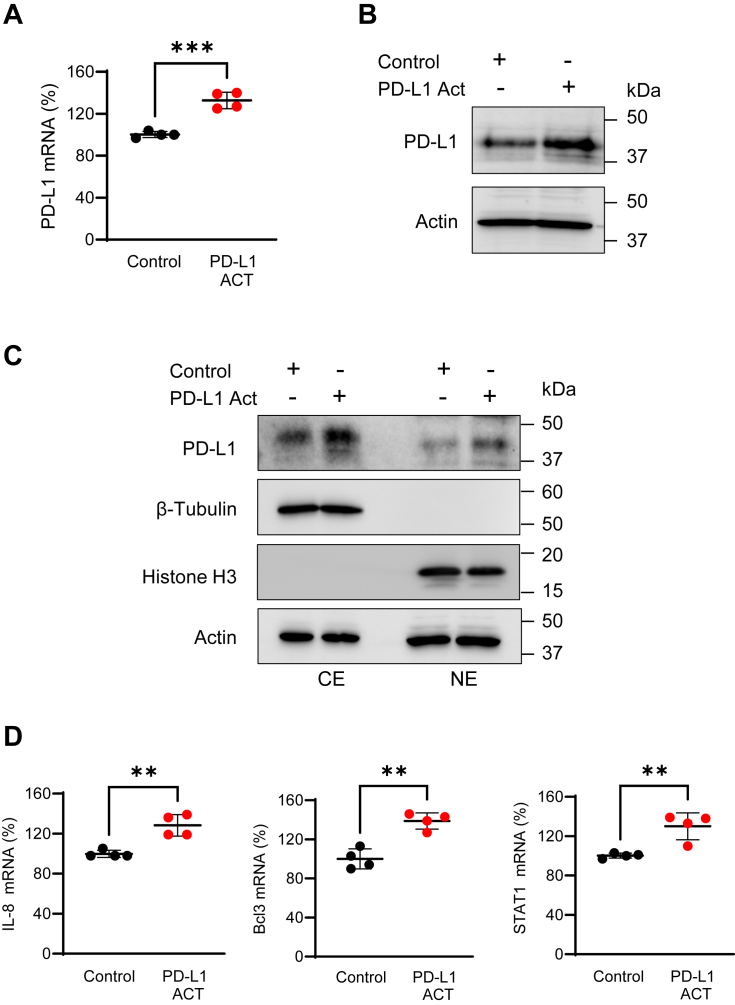
**Ectopic expression of PD-L1 induces nuclear localization of PD-L1 and expression of IL-8, Bcl3, and STAT1 in OC cells even in the absence of IFNγ.** (*A*) qRT–PCR of PD-L1 mRNA and (*B*) immunoblotting analysis of PD-L1 protein levels in WCE of unstimulated SKOV3 cells transfected with 3 μg of control plasmid (sc-437275) or PD-L1 activation plasmid (sc-401140-ACT). In the immunoblotting analysis, each lane represents approximately 10,000 cells; equal protein loading was confirmed by actin. *C*, immunoblotting analysis of PD-L1 in CE and NE extracts prepared from unstimulated SKOV3 cells transfected with 3 μg of control or PD-L1 activation plasmids as described previously. For CE, each lane represents approximately 15,000 cells, and for NE, 60,000 cells. The purity of cytoplasmic and nuclear fractions was monitored by using histone H3 and β-tubulin antibodies, respectively; equal protein loading was confirmed by actin. *D*, qRT–PCR analysis of IL-8, Bcl3, and STAT1 mRNA expression in unstimulated SKOV3 cells transfected with 3 μg of control or PD-L1 activation plasmids as described previously. Data are presented as the mean ± SD (n = 4). Statistical significance was evaluated by unpaired *t* test; ∗∗*p* < 0.01; ∗∗∗*p* < 0.001 compared with cells transfected with control plasmid. CE, cytoplasmic extract; IFNγ, interferon-γ; IL-8, interleukin-8; NE, nuclear extract; OC, ovarian cancer; qRT–PCR, quantitative RT–PCR; WCE, whole-cell extract.

Since a recent study has shown that PD-L1 overexpression induces nuclear localization of PD-L1 in lung cancer cells (15), we wanted to test whether ectopic expression of PD-L1 in unstimulated OC cells, which otherwise express only low levels of PD-L1, which is localized predominantly in the cytoplasm (Fig. 1), might induce nuclear localization of PD-L1. To this end, we transfected unstimulated SKOV3 cells with control or PD-L1 activation plasmids, and analyzed PD-L1 expression, its cytoplasmic–nuclear distribution, and the levels of PD-L1-regulated genes, IL-8, Bcl3, and STAT1. As shown in Figure 7, transfection with PD-L1 activation plasmid resulted in about 30% increase in PD-L1 mRNA (Fig. 7*A*) and a corresponding increase in PD-L1 protein levels (Fig. 7*B*). However, compared with IFNγ, which induces a significant increase in PD-L1 mRNA and protein levels in OC cells (36) (Figs. 1 and 3), the increase in PD-L1 expression induced by PD-L1 transfection in unstimulated cells was relatively modest (Fig. 7, *A* and *B*). Interestingly, even this ∼30% increase in PD-L1 expression in the absence of IFNγ was associated with an increased nuclear localization of PD-L1 (Fig. 7*C*). In addition, the ∼30% increase in PD-L1 expression in unstimulated transfected cells was associated with ∼30% increase in the expression of IL-8, Bcl3, and STAT1 (Fig. 7*D*). These results indicate that it is not IFNγ itself, but rather the IFNγ-induced PD-L1 overexpression, which leads to the translocation of a fraction of PD-L1 to the nucleus, where it induces transcription of IL-8, Bcl3, and STAT1.

### PD-L1 facilitates pol II recruitment to IL-8, Bcl3, and STAT1 promoters in IFN**γ**-stimulated OC cells

**Figure 8:**
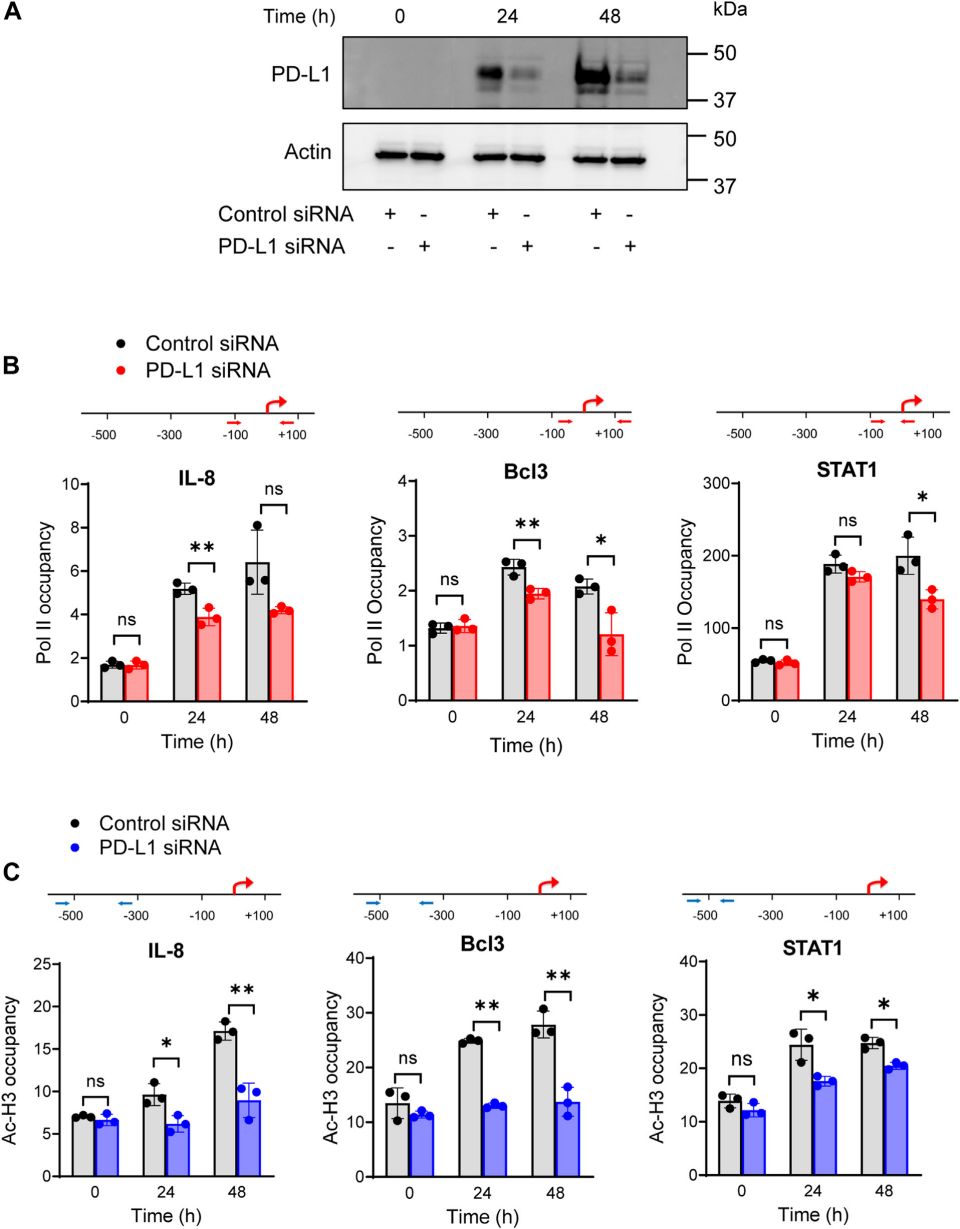
**PD-L1 facilitates pol II recruitment and promoter acetylation at IL-8, Bcl3, and STAT1 promoters in IFNγ-stimulated OC cells.***A*, Western blotting of SKOV3 cells transfected with PD-L1 or control siRNA and stimulated with 50 ng/ml IFNγ for 0, 24, and 48 h. Each lane represents approximately 10^4^ cells; equal protein loading was confirmed using actin antibody. *B* and *C*, ChIP in SKOV3 cells transfected with PD-L1 or control siRNA and stimulated with IFNγ (50 ng/ml) for 0, 24, and 48 h, using (*B*) pol II or (*C*) K9/14 acetylated histone H3 (ac-H3) antibodies. Purified DNA was analyzed by quantitative real-time PCR using designed primers spanning the TSS and ∼−500 bp regions in human IL-8, Bcl3, and STAT1 promoters (Table 1). The results are presented as a -fold difference in occupancy of pol II or Ac-H3 at the particular locus in comparison with the human IGX1A (SA Biosciences) locus, which does not contain any transcription factor–binding sites. Data are presented as the mean ± SD (n = 3). The results were analyzed by multiple unpaired *t* test; ∗*p* < 0.05; ∗∗*p* < 0.01; compared with cells transfected with control siRNA. ChIP, chromatin immunoprecipitation; IFNγ, interferon-γ; IL-8, interleukin-8; OC, ovarian cancer; pol II, polymerase II; TSS, transcription start site.

Since our data showed that PD-L1 regulates the mRNA levels of IL-8, Bcl3, and STAT1 in OC cells, we next investigated whether PD-L1 regulates transcription of these genes. Pol II recruitment to transcription start sites (TSSs) of gene promoters is a key step in transcriptional regulation; thus, using chromatin immunoprecipitation (ChIP), we measured pol II occupancy at TSS of IL-8, Bcl3, and STAT1 promoters in OC cells with downregulated PD-L1 expression (Fig. 8, *A* and *B*). In SKOV3 cells transfected with control siRNA, IFNγ increased pol II occupancy at IL-8, Bcl3, and STAT1 promoters (Fig. 8*B*); this is consistent with the enhanced expression of these genes in OC cells in response to stimulation with IFNγ (35, 36, 37). Importantly, transfection with PD-L1 siRNA decreased the pol II occupancy at IL-8, Bcl3, and STAT1 promoters in IFNγ-stimulated cells (Fig. 8*B*), indicating that PD-L1 induces transcription of IL-8, Bcl3, and STAT1 by facilitating the pol II recruitment.

### PD-L1 promotes IFN**γ**-induced IL-8, Bcl3, and STAT1 promoter acetylation

Since increased gene expression is associated with promoter acetylation, and because our recent studies have shown that IFNγ induces promoter acetylation in OC cells (35, 36, 37), we hypothesized that PD-L1 might regulate IFNγ-induced IL-8, Bcl3, and STAT1 expression by facilitating histone acetylation at their promoters. To this end, we analyzed K9/14 acetylated histone H3 (Ac-H3) occupancy at the promoters of IL-8, Bcl3, and STAT1 in cells with suppressed PD-L1 expression stimulated with IFNγ. Interestingly, when we used PCR primers spanning TSS of IL-8, Bcl3, and STAT1, as we used for the analysis of pol II promoter occupancy (Fig. 8*B*), we did not detect any increase in promoter acetylation in response to IFNγ stimulation (data not shown). However, using primers spanning the upstream regions located −500 to −300 bp from TSS, we observed a considerable increase in K9/14 Ac-H3 occupancy at IL-8, Bcl3, and STAT1 promoters in IFNγ-stimulated cells (Fig. 8*C*). Notably, the IFNγ-induced K9/14 Ac-H3 occupancy at IL-8, Bcl3, and STAT1 promoters was significantly decreased in cells with downregulated PD-L1 expression (Fig. 8*C*), indicating that PD-L1 promotes transcription of IL-8, Bcl3, and STAT1 by facilitating promoter histone acetylation and pol II recruitment.

### PD-L1 is recruited to IL-8, Bcl3, and STAT1 promoters in IFN**γ**-stimulated OC cells

**Figure 9:**
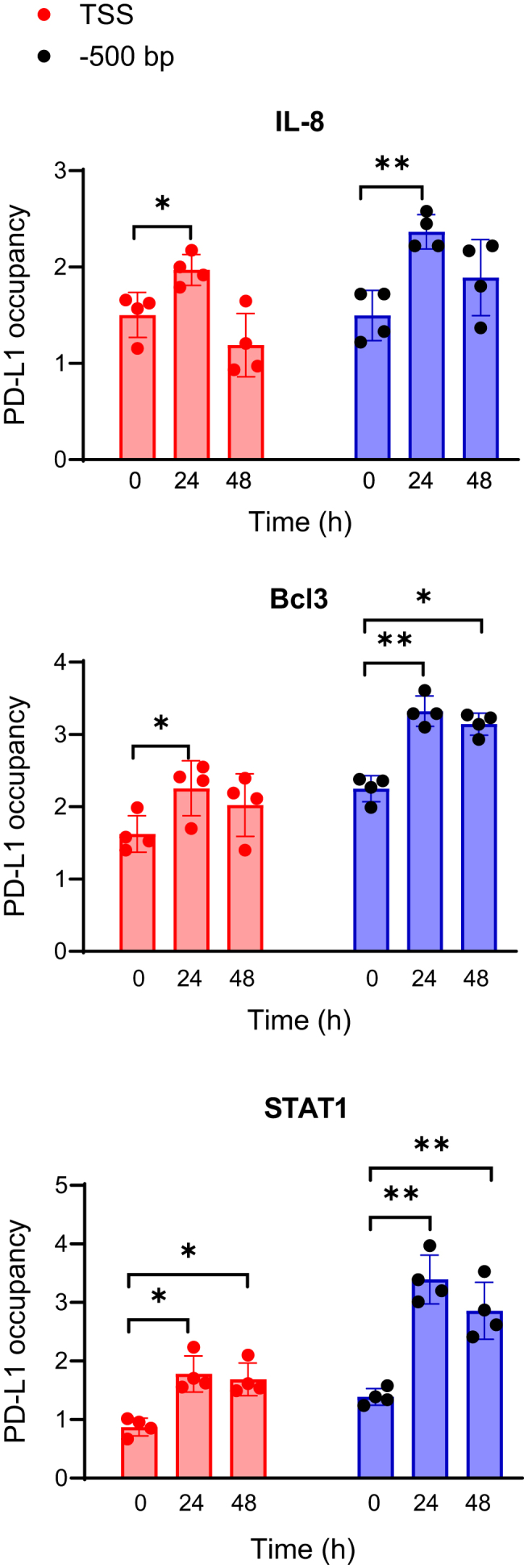
**PD-L1 is recruited to IL-8, Bcl3, and STAT1 promoters in IFNγ-stimulated OC cells.** ChIP analysis of PD-L1 recruitment to TSS and upstream (∼−500 bp) regions of human IL-8, Bcl3, and STAT1 promoters in IFNγ (50 ng/ml) stimulated SKOV3 cells. Purified DNA was analyzed by quantitative PCR using the primers listed in Table 1. The results are presented as a -fold difference in PD-L1 occupancy at the particular locus in comparison with the negative control IGX1A (SA Biosciences) locus. Data are presented as the mean ± SD (n = 4). The results were analyzed by ANOVA Tukey's *post hoc* test; ∗*p* < 0.05; ∗∗*p* < 0.01; compared with unstimulated cells (T = 0 h). ChIP, chromatin immunoprecipitation; IFNγ, interferon-γ; IL-8, interleukin-8; OC, ovarian cancer; TSS, transcription start site.

**Figure 10:**
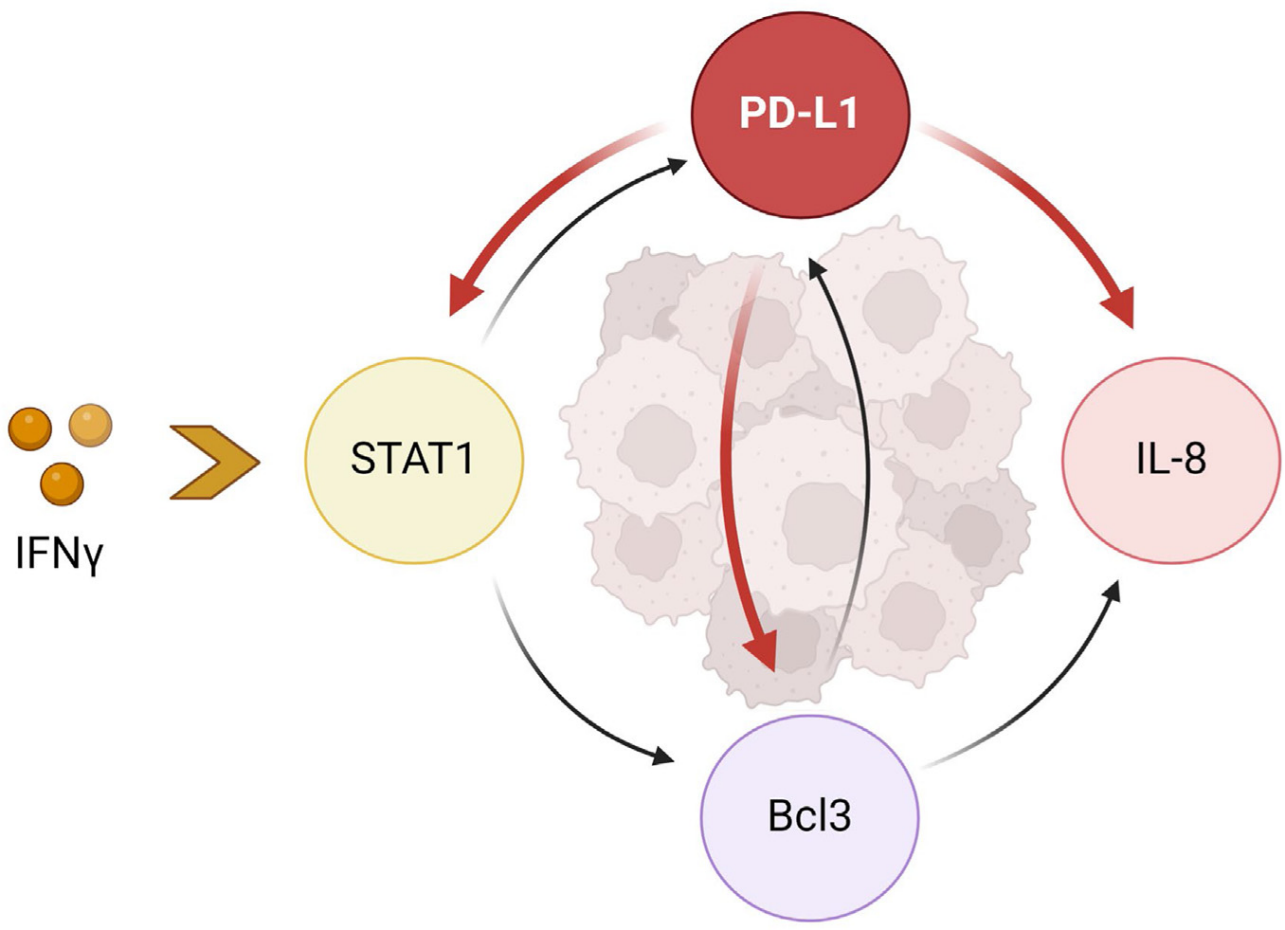
**Schematic model of the regulation of IL-8, Bcl3, and STAT1 expression by PD-L1 in IFNγ-stimulated OC cells**. IFNγ stimulates STAT1-dependent expression of Bcl3, PD-L1, and IL-8, resulting in increased proliferation of OC cells. The IFNγ-induced PD-L1 then stimulates transcription of IL-8, Bcl3, and STAT1, thus providing a positive feedback regulatory mechanism in IFNγ-stimulated cells. Created with BioRender.com with granted permission and license. IFNγ, interferon-γ; IL-8, interleukin-8; OC, ovarian cancer.

To investigate whether PD-L1 itself might be recruited to IL-8, Bcl3, and STAT1 promoters, we performed ChIP using the PD-L1 E1L3N antibody that recognizes endogenous PD-L1 and PCR primers that span both the TSS and the upstream (∼−500 bp) promoter regions. Even though the PD-L1 occupancy at IL-8, Bcl3, and STAT1 promoters was lower compared with pol II or Ac-H3 occupancy (shown in Figs. 8), 24 h IFNγ stimulation significantly increased PD-L1 recruitment to TSS sites of IL-8, Bcl3, and STAT1 promoters, compared with unstimulated cells (Fig. 9). Furthermore, the PD-L1 promoter occupancy was even more pronounced when we used primers spanning the −500 bp sites (Fig. 9). Since these upstream −500 bp acetylated promoter regions contain multiple binding sites for different transcription factors, these data suggest that PD-L1 might associate with DNA by binding to other transcription factors or regulators. Together, our findings suggest an interesting novel regulatory mechanism, in which IFNγ induces STAT1-dependent Bcl3, IL-8, and PD-L1 expression, and the induced PD-L1 further promotes the expression of IL-8, Bcl3, and STAT1 by increasing their transcription in IFNγ-stimulated cells (Fig. 10).

## Discussion

Nuclear localization of PD-L1 has been associated with cancer progression (21, 22, 23, 24, 25, 26, 27, 28), but the specific nuclear functions of PD-L1 are just emerging. We have recently shown that IFNγ induces STAT1-dependent expression of Bcl3, IL-8, and PD-L1 in OC cells, resulting in their increased proliferation and invasion (35, 36, 37). Here, we demonstrate that the IFNγ-induced PD-L1 associates with IL-8, Bcl3, and STAT1 promoters and facilitates histone acetylation and recruitment of pol II to the corresponding genes. The PD-L1-dependent activation of IL-8, Bcl3, and STAT1 promoters is associated with increased mRNA levels of IL-8, Bcl3, and STAT1 and decreased apoptosis in IFNγ-stimulated OC cells. To our knowledge, this is the first finding demonstrating that the immune checkpoint PD-L1 facilitates transcription by inducing promoter acetylation and pol II recruitment.

IFNγ is a pleiotropic cytokine produced not only by activated leukocytes but also in response to immune checkpoint blockade or radiation therapy used in cancer treatment (41, 42, 43, 44). Since early studies demonstrated numerous antitumor functions of IFNγ, including increased apoptosis of cancer cells, IFNγ has been used in cancer treatment (32, 33). However, most clinical trials using IFNγ in cancer treatment have produced disappointing results (45, 46, 47). Indeed, more recent studies have revealed also important tumor-promoting functions of IFNγ that include increased expression of immune checkpoints and enhanced cancer cell proliferation, but the specific mechanisms are not fully understood (9, 30, 31, 48, 49, 50). Our study shows that IFNγ induces nuclear localization of PD-L1 in OC cells (Fig. 1), and that suppression of the induced PD-L1 increases apoptosis (Fig. 2) and decreases expression of IL-8, Bcl3, and STAT1 (Figure 4, Figure 5, Figure 6). Since the increased expression of IL-8 and Bcl3, regulated by STAT1 in IFNγ-stimulated cells, increases survival and proliferation in OC cells (35, 36, 37), these results indicate that the IFNγ-induced PD-L1 inhibits apoptosis and enhances cancer cell proliferation by promoting the expression of IL-8, Bcl3, and STAT1. These findings suggest an interesting positive feedback regulatory mechanism, in which IFNγ induces STAT1-dependent Bcl3, IL-8, and PD-L1 expression, and the induced PD-L1 further promotes the expression of IL-8, Bcl3, and STAT1 by facilitating their transcription in IFNγ-stimulated cells (Fig. 10).

Interestingly, our data show that ectopic expression of PD-L1 induces PD-L1 nuclear localization in OC cells and upregulates expression of IL-8, Bcl3, and STAT1 even in the absence of IFNγ signaling (Fig. 7). These results indicate that it is not IFNγ itself, but the IFNγ-induced overexpression of PD-L1, that leads to the translocation of a portion of PD-L1 to the nucleus, where it induces transcription of IL-8, Bcl3, and STAT1. Similar results were observed by Du *et al.* (15) in lung cancer cells and HeLa cells, where ectopic PD-L1 overexpression induced translocation of a part of PD-L1 to the nucleus.

Even though recent studies have suggested PD-L1 role in transcriptional regulation, the specific mechanisms by which PD-L1 regulates transcription are largely unknown (12, 13, 14, 15, 51, 52, 53, 54). Our data, demonstrating that suppression of PD-L1 decreases pol II recruitment to IL-8, Bcl3, and STAT1 promoters and promoter acetylation in IFNγ-stimulated cells (Fig. 8), indicate that the IFNγ-induced nuclear PD-L1 facilitates promoter acetylation and pol II recruitment. Pol II transcription typically starts with pol II assembly with general transcription factors that form the preinitiation complex. The activity of pol II is highly regulated by interactions with different transcription factors and regulators; hundreds of factors have been identified to regulate pol II access to gene promoters and initiate transcription (55, 56). While some of the regulatory proteins can interact with pol II directly, others regulate gene expression by reorganizing nucleosomes and modifying chromatin, thus affecting the transcriptional status of pol II (55, 56). It will be important to identify the specific functions the nuclear PD-L1 has in regulating promoter acetylation and pol II recruitment in cancer cells.

PD-L1 does not possess a DNA-binding domain; however, several recent studies have shown that it can associate with different transcription factors and regulators, including STAT3, p65 NFκB, Sp1, and proteins involved in DNA damage response (12, 14, 15, 27, 57). Our data show that IFNγ-induced PD-L1 overexpression increases PD-L1 occupancy at IL-8, Bcl3, and STAT1 promoters, resulting in the increased expression of these genes. However, compared with pol II or acetylated histone H3 occupancy, the occupancy of PD-L1 was relatively low, suggesting that PD-L1 associates with these promoters indirectly, by binding to other transcriptional regulators. This is supported also by our results showing that for all three genes, the occupancy of PD-L1 was higher upstream (∼500 bp) of TSS, compared with the TSS sites (Fig. 9). Since the upstream regions of IL-8, Bcl3, and STAT1 promoters are rich in binding sites for several transcription factors, including NFκB, IRF1, STAT1, and STAT3, these data indicate that PD-L1 associates with the promoters and increases IL-8, Bcl3, and STAT1 transcription through associating with other transcriptional regulators.

In summary, our findings reveal a novel function of PD-L1, which consists of increased PD-L1 promoter occupancy, resulting in increased promoter acetylation and pol II recruitment, and increased expression of IL-8, Bcl3, and STAT1 in OC cells. The currently used PD-L1 blocking cancer immunotherapies target only the surface expression of PD-L1 but not its intracellular functions. Since the increased expression of IL-8 and Bcl3 increases proliferation and migration of OC cells (34, 35, 36, 37), our results suggest that the increased IL-8, Bcl3, and STAT1 expression mediated by IFNγ-induced nuclear PD-L1 might contribute to the limited effectiveness of the currently used PD-L1 blocking cancer immunotherapies.

## Experimental procedures

### Cell culture and transfections

OC SKOV3 and OVCAR3 cells were purchased from and authenticated by the American Type Culture Collection; they were cultured as described (34). Cells were tested for mycoplasma using the PCR-based Mycoplasma Detection Kit from the American Type Culture Collection (30–1012K). Cell viability was measured by trypan blue exclusion. Human recombinant IFNγ (285-IF-100; R&D Systems, Minneapolis, MN) was reconstituted in sterile water and used at a final concentration of 50 ng/ml (34).

Cells were transiently transfected with human PD-L1 CRISPR activation plasmid (sc-401140-ACT), control CRISPR activation plasmid (sc-437275), PD-L1-specific siRNA (sc-39699) or nonsilencing control siRNA (sc-37007) purchased from Santa Cruz Biotechnology according to the manufacturer’s protocols and as described previously (34, 35, 36, 37). Transfection efficiency was evaluated by both quantitative RT–PCR (qRT–PCR) and at a protein level by immunoblotting using PD-L1-specific monoclonal antibody E1L3N that recognizes endogenous PD-L1 (Cell Signaling Technology, catalog no.: 13684).

### Preparation of cytoplasmic and nuclear extracts

Cytoplasmic and nuclear extracts were prepared as described previously (58). Purity of nuclear and cytoplasmic fractions was determined by Western blotting using β-tubulin and histone H3 as specific cytoplasmic and nuclear markers, respectively. Equal protein loading was confirmed by using actin antibody.

### Western blotting

Protein samples were resolved by SDS-PAGE and transferred to Hybond ECL nitrocellulose membrane. The membranes were probed with primary antibodies against PD-L1 (Cell Signaling Technology, catalog no.: 13684; 1:1000 dilution), β-tubulin (Cell Signaling Technology, catalog no.: 2146; 1:1000 dilution), histone H3 (Cell Signaling Technology, catalog no.: 9715; 1:1000 dilution), Bcl3 (Proteintech, catalog no.: 23959-I-AP; 1:500 dilution), STAT1 (Cell Signaling Technology, catalog no.: 9172; 1:1000 dilution), and control actin (Sigma, catalog no.: A5060; 1:2000 dilution). After washing, the membranes were incubated with horseradish peroxidase–conjugated anti-rabbit immunoglobulin G (Novus Biologicals, catalog no.: NB7185; 1:5000 dilution), and the labeled proteins were detected using ECL Western Detection Reagent (Amersham) and Bio-Rad ChemiDoc imaging system.

### Apoptosis assay

Apoptosis was measured using a cell death detection ELISA kit that quantifies the release of nucleosomes into the cytoplasm (Cell Death Detection ELISAPLUS kit; Roche) as described (59).

### Real-time qRT–PCR

Total RNA was isolated using RNeasy mini-kit (Qiagen), and qRT–PCR was performed as described (34, 35, 36, 37). All values were normalized using actin. The primers for quantification of human PD-L1, IL-8, Bcl3, STAT1, and actin mRNA were from Qiagen. The mRNA values are expressed as a percentage compared with untreated cells or cells transfected with control siRNA.

### Chromatin immunoprecipitation

ChIP was performed as described (34, 35), using the following antibodies: pol II (Santa Cruz Biotechnology; catalog no.: sc-56767), PD-L1 (Cell Signaling Technology, catalog no.: 13684), Lys9 Ac-H3 (Cell Signaling Technology, catalog no.: 9649S), and control immunoglobulin G (Cell Signaling Technology, catalog no.: 3900). Each immunoprecipitation was performed at least three times using different chromatin samples. Purified DNA was analyzed by real-time qPCR using designed primers spanning the TSS and ∼−500 bp regions of human IL-8, Bcl3, and STAT1 promoters (Table 1). The promoter occupancy was calculated as a fold difference in RNA pol II, Ac-H3, and PD-L1 occupancy at IL-8, Bcl3, and STAT1 promoters compared with control negative human IGX1A locus (Qiagen; catalog no.: 334001) that does not bind any transcription factors.

**Table 1:** qPCR primers used for ChIP

Gene	5′- start from TSS	Primer	Sequence
*IL-8*: TSS	−113	Forward	5′-TGGGCCATCAGTTGCAAATC-3′
	+80	Reverse	5′-AGTGAGATGGTTCCTTCCGG-3′
*IL-8*: Upstream	−534	Forward	5′-TTTGAAAAGTTGTAGTATGCCCC-3′
	−321	Reverse	5′-AGAGTGGCAGGTGTTAGAAC-3′
*Bcl3*: TSS	−30	Forward	5′-GACAAAAGTCCCTTCAGTTCAGC-3′
	+124	Reverse	5′-ACGGGCCCCTCGTCCAT-3′
*Bcl3*: Upstream	−505	Forward	5′-AACTGAGAGGCAGAGAGATG-3′
	−312	Reverse	5′-CTGCCTCTGTTTTTGTCTTT-3′
*STAT1*: TSS	−97	Forward	5′-AACAGCCGCGTCTAATTG-3′
	+14	Reverse	5′-CAGGAAAGCGAAACTACCC-3′
*STAT1*: Upstream	−569	Forward	5′-TGGAGGTGGAGGCAATGTAG-3′
	−456	Reverse	5′-GCAGAGTTGACGCCTTTGTT-3′

### ELISA

Release of human IL-8–CXCL8 into cell culture supernatants was measured by ELISA (R&D Systems; catalog no.: D8000C).

### Statistical analysis

All experiments were performed at least three times, analyzed in duplicates, and expressed as means ± SD. All statistical analyses (unpaired *t* test, ANOVA, and tests for normal distribution of data and statistical outliers) were performed in GraphPad Prism 10.4.1 package (GraphPad Software, Inc). Statistical significance between two groups was evaluated using unpaired *t* test and between three or more groups using ANOVA Tukey's *post hoc* test; *p* < 0.05 was considered significant.

## Data availability

All data that support this study are provided in the article. Further inquiries can be directed at the corresponding author.

## Conflict of interest

The authors declare that they have no conflicts of interest with the contents of this article.
